# Morphological Variations within the Ontogeny of *Deinonychus antirrhopus* (Theropoda, Dromaeosauridae)

**DOI:** 10.1371/journal.pone.0121476

**Published:** 2015-04-15

**Authors:** William L. Parsons, Kristen M. Parsons

**Affiliations:** Buffalo Museum of Science, Department of Geology, 1020 Humboldt Parkway, Buffalo, New York, United States of America; University of Pennsylvania, UNITED STATES

## Abstract

This research resulted from the determination that MCZ 8791 is a specimen of *Deinonychus antirrhopus* between one and two years of age and that the morphological variations within particular growth stages of this taxon have yet to be described. The primary goal of the research is to identify ontogenetic variations in this taxon. Histological analyses determined that the *Deinonychus* specimens AMNH 3015 and MOR 1178 were adults. Comparisons are made between MCZ 8791 and these adult specimens. The holotype, YPM 5205, and the other associated specimens of this taxon within the YPM collection are similar in size and morphology to AMNH 3015. Further comparisons were made with the three partial specimens OMNH 50268, MCZ 4371, and MOR 1182. Although these specimens represent only a partial ontogenetic series, a number of morphological variations can be described. One secondary goal of this research is to compare the known pattern of variable, informative, ontogenetic characters in MCZ 8791 to a similar pattern of morphological characters in the sub-adult dromaeosaurid specimen *Bambiraptor feinbergorum*, AMNH FR: 30556. If the characters that have been determined to represent variable juvenile morphology in the ontogeny of *Deinonychus* are exhibited in *Bambiraptor*, this study will begin the process of determining whether a similar, conservative, ontogenetic pattern exists throughout the rest of Dromaeosauridae. If defensible, it may reduce the number of sympatric taxa within this clade. The other secondary goal relates to the forelimb function. The approximate body size, forelimb length, wrist development, and the presence of a more prominent olecranon on the ulna of MCZ 8791 support the hypothesis that juveniles of this taxon possessed some form of flight capability.

## Introduction

The dromaeosaurid *Deinonychus antirrhopus* [1] is known from several specimens collected from the Early Cretaceous Cloverly Formation of Montana [1–3] and one specimen from the Antlers Formation of Oklahoma, Oklahoma Museum of Natural History (OMNH) 50268 [4]. The growth stages of the four Yale/Peabody Museum of Natural History (YPM) specimens were not determined in Ostrom’s original description. OMNH 50268 was determined by Brinkman et al. [4] to be a sub-adult. *D*. *antirrhopus* was a medium-sized member of the clade Dromaeosauridae, which possesses an important evolutionary relationship to Avialae.

In 1982, a partial skeleton of a small theropod, Museum of Comparative Zoology (MCZ) 8791, was recovered from an Early Cretaceous, Cloverly Formation site in central Montana. It was collected by Charles Schaff from the Museum of Comparative Zoology at Harvard University. Recently, two incomplete coracoids and the right manual II-2 phalanx from MCZ 8791 have been prepared and identified.

### Identification of MCZ 8791

**Fig 1:**
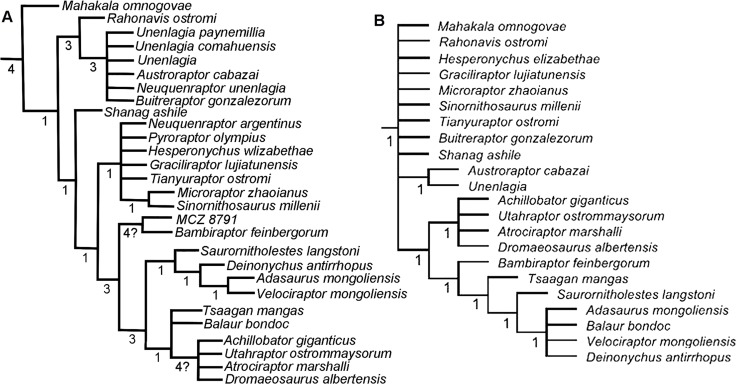
Detailed portions of two consensus tree cladograms. **A**, Detail of the dromaeosaurid portion of the paravian section of the strict consensus cladogram generated by the cladistic analysis and Bremer support resampling of the entire Turner et al. (2012) data matrix with the addition of MCZ 8791; **B**, Detail of the dromaeosaurid portion of the paravian section of the strict consensus cladogram generated by the cladistic analysis and Bremer support resampling of the entire Turner et al. (2012) data matrix without MCZ 8791. Values indicate Bremer support. Both analyses used TNT version 1.0 software [6].

Sixty-two phylogenetic characters have been identified from MCZ 8791 (S1 Supporting information). This specimen and its characters were added to the Turner et al. data matrix [5]. From this newly edited data matrix, a strict consensus tree from 59 retained trees using a TNT software traditional search format [6] was generated. The support strength of the branching within this consensus tree was measured using Bremer support resampling. Fig 1A presents a detail of the complete, new tree with the Bremer support data, representing the dromaeosaurid branching including MCZ 8791 in the data matrix. For comparative purposes, Fig 1B is directly cited from Turner et al. [5]. In this current analysis, the best score (the tree with the least steps) was 1,989 steps. This best score was hit 3 times out of ten. The results of this analysis were further checked by conducting a similar heuristic search analysis on the same data matrix with PAUP cladistic software [7]. The best tree length was again 1,989 steps, and 1,000 trees were retained. The results of the PAUP consensus tree analysis were the same as the results from the TNT analysis. The consistency index excluding uninformative characters equaled 0.3028. The homoplasy index excluding uninformative characters equaled 0.6972. The retention index equaled 0.7325. The rescaled consistency index equaled 0.2247. When MCZ 8791 is added to the Turner et al. data matrix, there is some improvement to the hierarchal resolution within the taxa in Dromaeosauridae, although the overall branching is still weakly supported at many levels. MCZ 8791 and *Bambiraptor feinbergorum* were resolved into a sister taxa branch (Fig 1). MCZ 8791 falls out basal to *D*. *antirrhopus*. This basal positioning of MCZ 8791 is similar to the results of cladistic analyses of other juvenile dinosaur specimens. Given the far more complete character set obtained from the adult specimens of *D*. *antirrhopus*, the number of shared derived characters between *D*. *antirrhopus* and other more completely known members of Dromaeosauridae shifts the placement of *D*. *antirrhopus* away from that of MCZ 8791. The sister taxa branching between MCZ 8791 and *B*. *feinbergorum* is due to both the limited number of characters available to describe MCZ 8791 and a consequence of both being sub-adults within their respective taxa. Neither possesses the derived characters that only develop closer to the adult growth stages. The most relevant result of this analysis is that all the sixty-two known characters in MCZ 8791 are synapomorphic to the same characters identified by Turner et al. in *D*. *antirrhopus* [5]. These characters are listed in S1 Character List.

In the phylogenetic analyses of other coelurosaurid theropods [8] as well as ornithischian dinosaurs [9], other problematic cladistic placements of juvenile specimens have been observed. Certain specimens from early growth stages of other theropod taxa code out as basal to the known taxon due to the many derived characters that only appear ontogenetically close to the adult forms [5]. In comparison to known adult specimens, the analysis of Mongolian Paleontological Center (MPC)-D 107/7, a juvenile specimen of *Tarbosaurus bataar*, resulted in a phylogenetic placement that was basal to the adult specimens [8].

**Fig 2:**
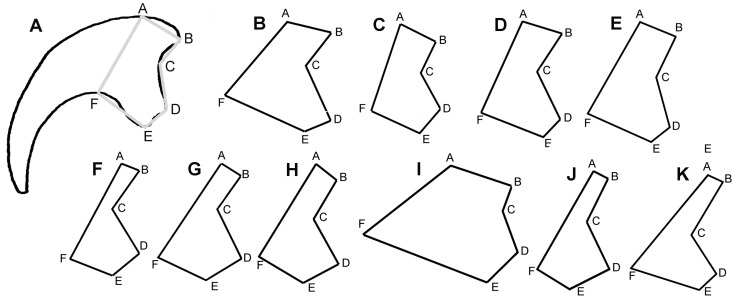
Shape-graphing of dromaeosaurid second pedal unguals. **A**, outline of *D*. *antirrhopus* second pedal ungual YPM 5205 with designated landmark points and simplified internal representation of the morphology; **B**, *B*. *feinbergorum* AMNH FR 30554 [10,11]; **C**, *D*. *antirrhopus* specimen YPM 5205 [1]; **D**, *D*. *antirrhopus* specimen MCZ 8791; **E**, *D*. *antirrhopus* specimen OMNH 50268 (from cast YPM 55845); **F**, *N*. *argentinus* MCF PUPH 77 [12]; **G**, *H*. *elizabethae* UALVP 48778 [13]; **H**, *R*. *ostromi* UA 8656 [14]; **I**, *U*. *ostrommaysorum* CEU 184v.86 [15]; **J**, *V*. *mongoliensis* IGM 100/985 (pers. obs.); **K**, *B*. *bondoc* EME PV.313 [16].

Along with these 62 synapomorphic characters, the best further evidence for the referral of the juvenile specimen MCZ 8791 to *D*. *antirrhopus* comes from an analysis of the morphology of the dentition and the lateral profile of the second pedal ungual. The one articulated maxillary tooth of MCZ 8791 possesses 18.5 distal serrations per 5 mm. Within Ostrom's table of theropod tooth serration variation [1], this is closest to the 16 to 18 distal serrations described for *D*. *antirrhopus* [1]. Certain specific measurements between identical landmark points along the lateral profile of dromaeosaurid second pedal unguals create simplified internal representations of the morphology of these unguals (Fig 2). With the exception of the development of the flexor tubercle, which will later be described as an informative, variable, ontogenetic character, the ontogenetic development of the second pedal ungual represented by MCZ 8791, OMNH 50268, and YPM 5205 is isometric. A comparison of the morphologies of the dromaeosaurid second pedal ungual of *B*. *feinbergorum* American Museum of Natural History (AMNH) FR 30554 [10,11], *D*. *antirrhopus* YPM 5205 [1], *D*. *antirrhopus* MCZ 8791, *D*. *antirrhopus* OMNH 50268 (from cast YPM 55845), *Neuquenraptor argentinus* Museo Carmen Funes (MCF) PUPH 77 [12], *Hesperonychus elizabethae* University of Alberta Laboratory for Vertebrate Palaeontology (UALVP) 48778 [13], *Rahonavis ostromi* Université d’Antananarivo (UA) 8656 [12], *Utahraptor ostrommaysorum* College of Eastern Utah Prehistoric Museum (CEU) 184v.86 [13], *Velociraptor mongoliensis* Mongolian Institute of Geology (IGM) 100/985 (pers. obs.), and *Balaur bondoc* Transylvanian Museum Society, Dept. of Natural Sciences (EME) PV.313 [14] (Fig 2) displays a considerable variation in the morphology of this ungual throughout Dromaeosauridae. The similarity in morphology of the lateral profile of the MCZ 8791 second pedal ungual to that of other members of *D*. *antirrhopus* is diagnostic to this taxon (Fig 2).

### Age assessment of MCZ 8791

**Fig 3:**
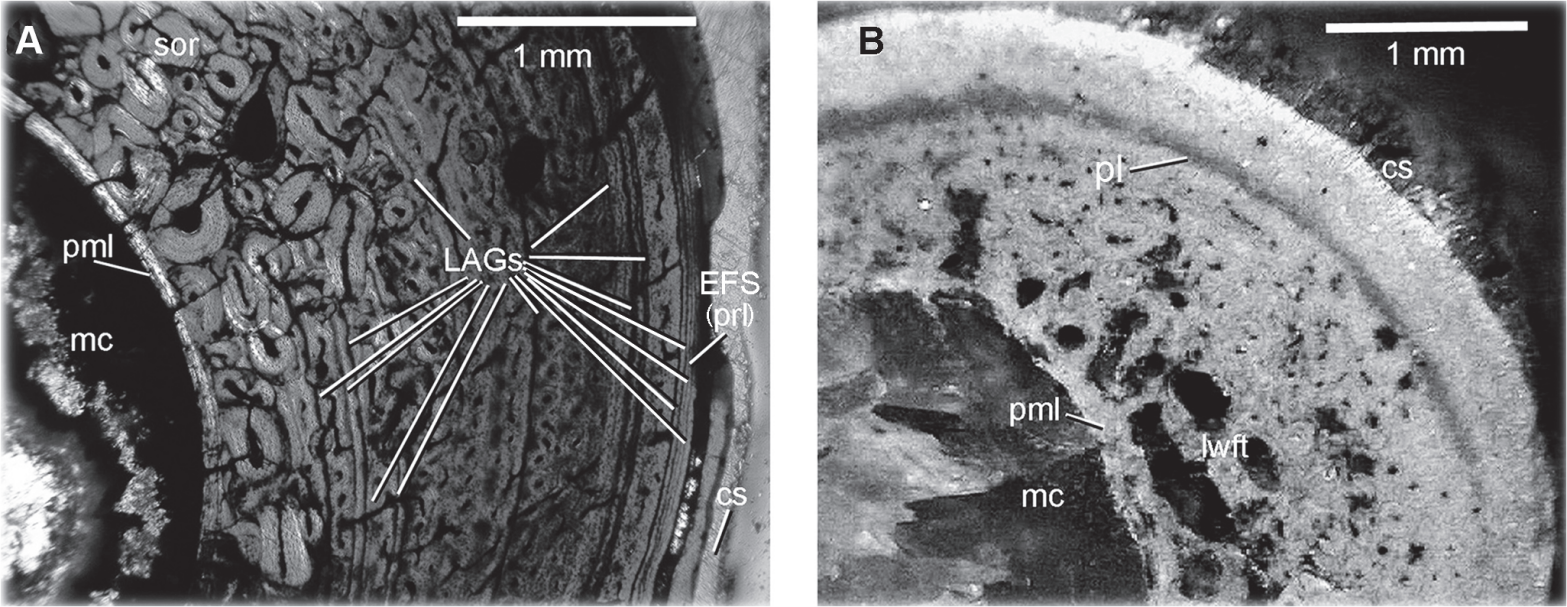
Histology of the radius of *D*. *antirrhopus*. **A**, histology from within a thin section of the radius of *D*. *antirrhopus* specimen MOR 1178; **B**, histology from within a polished cross-section of the radius of *D*. *antirrhopus* specimen MCZ 8791. All photographs of MOR *D*. *antirrhopus* specimens within this article are published with permission of Museum of the Rockies. All photographs of MCZ *D*. *antirrhopus* specimens within this article are published with permission of Museum of Comparative Zoology. **Abbreviations**: **cs**, cortical surface; **EFS**, External Fundamental System; **LAG**, Line of Arrested Growth; **lwft**, ‘loosely woven’ fibrolamellar tissue; **mc**, medullary cavity; **pl**, polish line; **pml**, perimedullary layer; **prl**, periosteal rest layers; **sor**, secondary osteon replacement layer.

Age assessment was determined through bone histology, specifically the examination of Lines of Arrested Growth (LAGs) or polish lines [17–22]. The histology of the radius of MCZ 8791 was compared to that of the radius of Museum of the Rockies (MOR) 1178 (Fig 3A and 3B). Within a polished cross-sectional surface of MCZ 8791 there is one polish line present (Fig 3B). Polish lines are considered the equivalent of LAGs [22]. This polished cross-section lacks any External Fundamental System (EFS), which is a tightly condensed grouping of LAGs in the periosteal region indicating a termination in the growth process, determinate growth, and thus adult growth status [23–29]. The one polish line indicates that the age of MCZ 8791 is between one and two years of age.

In order to obtain a complete sequence of LAGs for this research, retrocalculation was used to determine the completeness of the LAG sequence in MOR 1178 [29–31]. The measurement from the center point of the medullary cavity to the one polish line within the radius of MCZ 8791 determined where the location of the first MOR 1178 LAG should be found. The difference in width between the diameter of the MCZ 8791 medullary cavity and the diameter of the medullary cavity of the MOR 1178 radius represents the original juvenile bone matrix and LAGs that might have been resorbed during the ontogenetic expansion of the medullary cavity [32]. The diameters of the ovoid medullary cavity of MCZ 8791 are between 1.65 mm and 1.46 mm. Those of MOR 1178 are between 2.54 mm and 2.20 mm. The differences in the lengths of the two diameters of these two radii are respectively. 89 mm and. 74 mm. This indicates there was some ontogenetic expansion of the medullary cavity. The diameter of the complete circumferential polish line in the MCZ 8791 radius is between 2.73 mm and 2.60 mm. This places the potential first LAG within the region of bone matrix that was still retained within MOR 1178 (Fig 3A), and thus no earlier LAGs in the radius of MOR 1178 were lost due to the expansion of the medullary cavity. The endosteal region of the MOR 1178 radius retained fourteen LAGs. Given the final three LAGs occur within the EFS, this is a complete ontogenetic sequence of LAGs. A reduction in annual growth occurred at eleven years, and a termination of any increase in size due to determinate growth in MOR 1178 occurred at fourteen years.

Closer to the medullary cavity in the radius of MCZ 8791, there is highly vascularized, loosely woven, fibrolamellar tissue. This tissue possesses randomly arranged, large, globular lacunae of osteocytes. This is typical of the rapid bone growth in juvenile dinosaurs [31] (Fig 3B). The perimedullary layer separating the medullary cavity from the bone matrix is undifferentiated from the adjacent, amorphous, fibrolamellar tissue. In the adult MOR 1178 the differentiation is quite clear. These large, globular lacunae of osteocytes and the undifferentiated perimedullary layer are characters of the juvenile condition in this taxon as is exemplified in the juvenile MCZ 8791 (Fig 3B).

### Determination of adults within this taxon

Histological analysis of the *D*. *antirrhopus* specimen AMNH 3015 has revealed an EFS within the gastralia and dorsal ribs [32]. The presence of an EFS is evidence of determinate growth and thus the adult growth status of AMNH 3015. The histologically determined age of 13 to 14 years for MOR 1178 concurs with the age of maturity as determined for *D*. *antirrhopus* by Erickson et al. [32] (Fig 3A). MOR 1182, the YPM holotype material and associated material are comparable in size and morphology to AMNH 3015 [1] and thus are considered similar in age.

### Overall aim

Several ontogenetic variations in *D*. *antirrhopus* have been examined. A comparative analysis between the juvenile MCZ 8791, the sub-adult OMNH 50268, and adults of this taxon will focus on describing these ontogenetic variations. This research begins the process of describing ontogenetically diagnostic characters within Dromaeosauridae. The presence of a number of these informative ontogenetic characters in other members of Dromaeosauridae would indicate that an ontogenetically conservative pattern is shared between some members of Dromaeosauridae. This would be similar to the sharing of a conservative ontogenetic pattern in tyrannosaurids [33].

## Material and Methods

### Referred materials

*D*. *antirrhopus* specimens studied include: MCZ 4371, MOR 747, MOR 1178, MOR 1182, OMNH 50268, YPM 5203, YPM 5204, YPM 5205, YPM 5206, YPM 5207, YPM 5209, YPM 5210, YPM 5211, YPM 5217, YPM 5218, YPM 5220, YPM 5230, YPM 5232, YPM 5236, YPM 55845, and AMNH 3015 as per Ostrom 1969. Other theropod and avian specimens studied included: *Accipiter gentilis*, Buffalo Museum of Science (BMS) 7608; *B*. *bondoc* EME PV.313; *B*. *feinbergorum*, AMNH FR 30554; *Bubo virginiensis* BMS 8039; *Changyuraptor yangi*, Paleontological Center, Bohai University (HG) B016; *Glaucomys volans* BMS 1178; *H*. *elizabethae*, UALVP 48778; *Melanerpes erythrocephalus* BMS 914; *Microraptor gui*, Institute of Vertebrate Paleontology and Paleoanthropology (IVPP) V13352; *Microraptor zhaoianus*, IVPP V 12330; *N*. *argentines* MCF PUPH 77; *Pelagornis chilensis*, Museo Nacional de Historia Naturl (MNHN) SGO PV 1061; *R*. *ostromi* UA 8656; *T*. *bataar*, MPC-D 107/7; *U*. *ostrommaysorum* CEU 184v.86; and *V*. *mongoliensis* specimens, IGM 100/986, IGM 100/985.

### Material from sub-adult and juvenile specimens of *D*. *antirrhopus*

MCZ 8791 is a partial skeleton that includes a fragmentary left maxilla, one complete and two fragmentary maxillary teeth, a complete right articular, one cervical vertebra, five dorsal vertebrae, three proximal caudal vertebrae, three mid-caudal vertebrae, two partial (left and right) coracoids, proximal and distal ends of the right ulna, a proximal end of the left radius, a complete manual II-2 phalanx, an ilium fragment with an ischiadic peduncle, a fragment of the distal end of the femur, a partial tibia, a proximal end of fibula, a proximal end of the right pedal II-1 phalanx, a proximal end of the right pedal IV-4 phalanx, a proximal end of the left pedal IV-1 phalanx, and a partial right pedal II-3 ungual. All MCZ 8791 vertebrae are fragmentary. No neural arches are present. All the dorsal vertebrae retain a neurocentral sutural rugosity. Where fragmentation allows examination, all centra are hollow.

OMNH 50268 [4] is a small sub-adult specimen of *D*. *antirrhopus* from the Antlers Formation of Oklahoma. Only a few bones of this specimen are replicated by those of MCZ 8791. These are the right coracoid, the right pedal II-3 ungual, the left pedal IV-4 phalanx, and the maxillary teeth.

### Histology

Both a polished cross-section of the left radius of MCZ 8791 and a transverse thin-section from a mid-shaft fragment of the radius of the *D*. *antirrhopus* specimen MOR 1178 were prepared at the Buffalo Museum of Science (Buffalo, NY). The thin-section from the radius of MOR 1178 was mounted on a glass slide. Both the thin-section and the polished cross-sectional end of a mid-shaft fragment of the radius of MCZ 8791 were prepared with successive grades of silicon carbide up to 1200 grit and then washed.

### Phylogenetic protocol

The 62 phylogenetic characters aof MCZ 8791 were added to the entire data matrix of Turner et al. [5]. The original Turner et al. data matrix [5] was obtained from Morphobank (www.morphobank.org). The entire data matrix was initially analyzed using TNT (Trees using New Technology) version 1.0 software [6], traditional technology search. The strength of the support of the branches was determined through a Bremer support resampling analysis. The entire TNT analysis was rechecked by applying PAUP 4.0b10 for 32-bit Microsoft Windows [7] to the same data matrix using the same analytic processes. The phylogenetic character list for MCZ 8791 is in S1 Character List.

### Permits

No permits were required for the described study, which complied with all relevant regulations. All specimens discussed have been accessioned into duly accredited vertebrate paleontology collections. These specimens are all freely available for public research and examination.

## Results

### Locality/Formation/Age for MCZ 8791

MCZ 8791 was collected from the Pryor Mountain field near Wolf Creek (Red Creek) Se 1/4S. 14, T. 4S. R24E, Carbon County, Montana. The site locality for this specimen is in Unit 6, the middle unit of Units 5, 6, and 7 of the Himes member of the Early Cretaceous Cloverly Formation. This formation has been designated as Aptian/Albian age [1].

### Description of variable ontogenetic characters

Characters possessed by the juvenile specimens MCZ 8791 and/or OMNH 50268 that variably transform or disappear by the adult stage within the ontogeny of this taxon are defined as variable ontogenetic characters. These are the “characters” that are described in the following anatomical descriptions section. At some future point in this type of ontogenetic research, once all the variable ontogenetic characters within all the complete growth series of the taxa within a given clade have been determined, the inclusion of such characters within the phylogenetic analysis of that clade should contribute to further resolutions of the cladistic placements of the taxa within that clade. Although currently, we are dealing with only an incomplete assemblage of specimens from the ontogenetic stages within *D*. *antirrhopus*, a number of progressive morphological changes can be described.

### Description: skull of MCZ 8791

#### Right maxilla (Fig 4)

**Fig 4:**
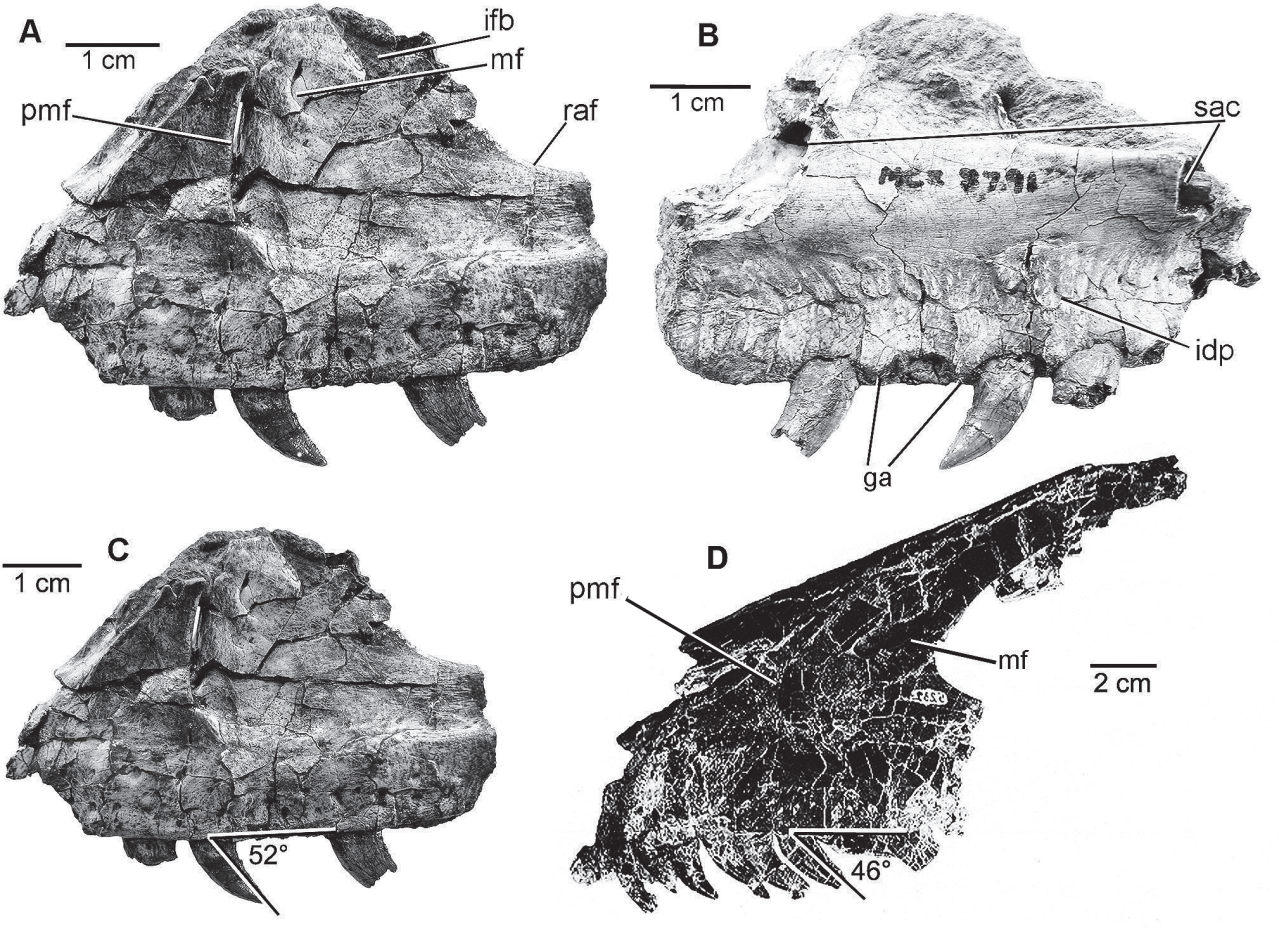
Maxilla of *D*. *antirrhopus*. Fragment of left maxilla on *D*. *antirrhopus* specimen MCZ 8791: **A**, MCZ 8791 lateral view; **B**, MCZ 8791 medial view; **C**, angle of tooth orientation in MCZ 8791; **D**, angle of tooth orientation in left maxilla of YPM 5232. **Abbreviations**: **ga**, gap between alveoli; **idp**, interdental plates; **ifb**, interfenestral bar; **mf**, maxillary fenestra; **pmf**, promaxillary fenestra; **raf**, rim of internal antorbital fenestra; **sac**, rostral and caudal ends of supra-alveolar canal. Photographs of MCZ 8791 courtesy of Museum of Comparative Zoology, Harvard University. All photographs and illustrations of YPM *D*. *antirrhopus* material within this article are published with permission of Yale Peabody Museum of Natural History.

A partial right maxilla was recovered (Fig 4A–4C). In order to conduct a comparative study of cranial proportions, a standard measurement was created from an intact portion of the maxillary tooth row. The distance between the mesial end of the alveolus directly beneath the ventral end of the promaxillary fenestra and the distal end of the alveolus of the third tooth behind that originating mesial point of measurement has been determined on both the right maxilla of MCZ 8791 (2.32 cm) and the left maxilla of YPM 5232 (4.59 cm). These measurements have been used to construct ratios that exhibit the proportional differences between the juvenile and adult maxillae.

The ratio between the vertical height of the promaxillary fenestra and our standardized three alveoli measurement is. 38 in MCZ 8791 and. 45 in YPM 5232. In the character of the vertical height of the promaxillary fenestra, the juvenile condition is shorter in contrast to length in the adult condition which is comparatively enlarged. The ratio of the distance between ventral end of the vertical slit-like promaxillary fenestra in MCZ 8791 and the ventral edge of the maxilla to the three alveoli measurement is. 67 as compared to. 68 in YPM 5232. In the character of the distance between the ventral end of the promaxillary fenestra and the ventral edge of the maxilla, the juvenile condition in MCZ 8791 is only slightly less in contrast to the height in the YPM 5232 adult condition.

The ratio created from the distance between the caudal edge of the promaxillary fenestra and the rostral edge of the antorbital fenestra and our standardized three alveoli measurement is. 20 in MCZ 8791 and. 32 in YPM 5232. In the character of the distance between the caudal edge of the promaxillary fenestra and the rostral edge of the antorbital fenestra, in the MCZ 8791 juvenile condition, this length is relatively less in contrast to the length in the YPM 5232 adult condition. These ratios indicate that the MCZ 8791 maxilla was comparatively shorter than the YPM maxilla.

The MCZ 8791 maxillary fenestra is a smaller, slit-like opening with a vertically oriented long axis. It is immediately caudal to the dorsal end of the promaxillary fenestra. In the character of the orientation of the long axis of the maxillary fenestra, the MCZ 8791 juvenile condition is vertically oriented; in contrast, the horizontal orientation of the long axis is the YPM 5232 adult condition.

In MCZ 8791, medially above the alveoli, there is a series of interdental plates (Fig 4B). In the character of the presence or absence of interdental plates, the MCZ 8791 juvenile condition has interdental plates present. In the YPM 5232 adult, the interdental plates are co-ossified to the extent that they cannot be distinguished from the medial alveolar process.

The ratio created from the rostral most point on the edge of the antorbital fenestra and the ventral edge of the maxilla and our standardized three alveoli measurement is. 83 in MCZ 8791 and 1.03 in YPM 5232. In the character of the height between the edge of the antorbital fenestra and the ventral edge of the maxilla, the MCZ 8791 juvenile condition is shorter, in contrast to the greater height which is the adult condition in the YPM 5232 maxilla. In the character of the angle between the rostrodorsal and ventral margins of this alveolar region that tapers in a caudal direction, the MCZ 8791 juvenile condition is an angle of 29°; in contrast, the angle in the adult YPM 5232 maxilla is 40°. The juvenile condition in MCZ 8791 is a diminished dorsoventral height of the alveolar region in MCZ 8791. The greater height in YPM 5232 is the adult condition.

#### Right articular (Figs 5 and 6)

A complete right articular of MCZ 8791 was recovered. In order to conduct a comparative study of the proportions of the articular, a standard measurement of length of the articular in medial view, from the contact area for the prearticular to the area of insertion for the M. depressor mandibular, has been determined on both the right articular of MCZ 8791(2.33 cm) and the right articular of YPM 5232 (3.60 cm). These measurements have been used to construct ratios that exhibit the proportional differences between the juvenile and adult articular.

**Fig 5:**
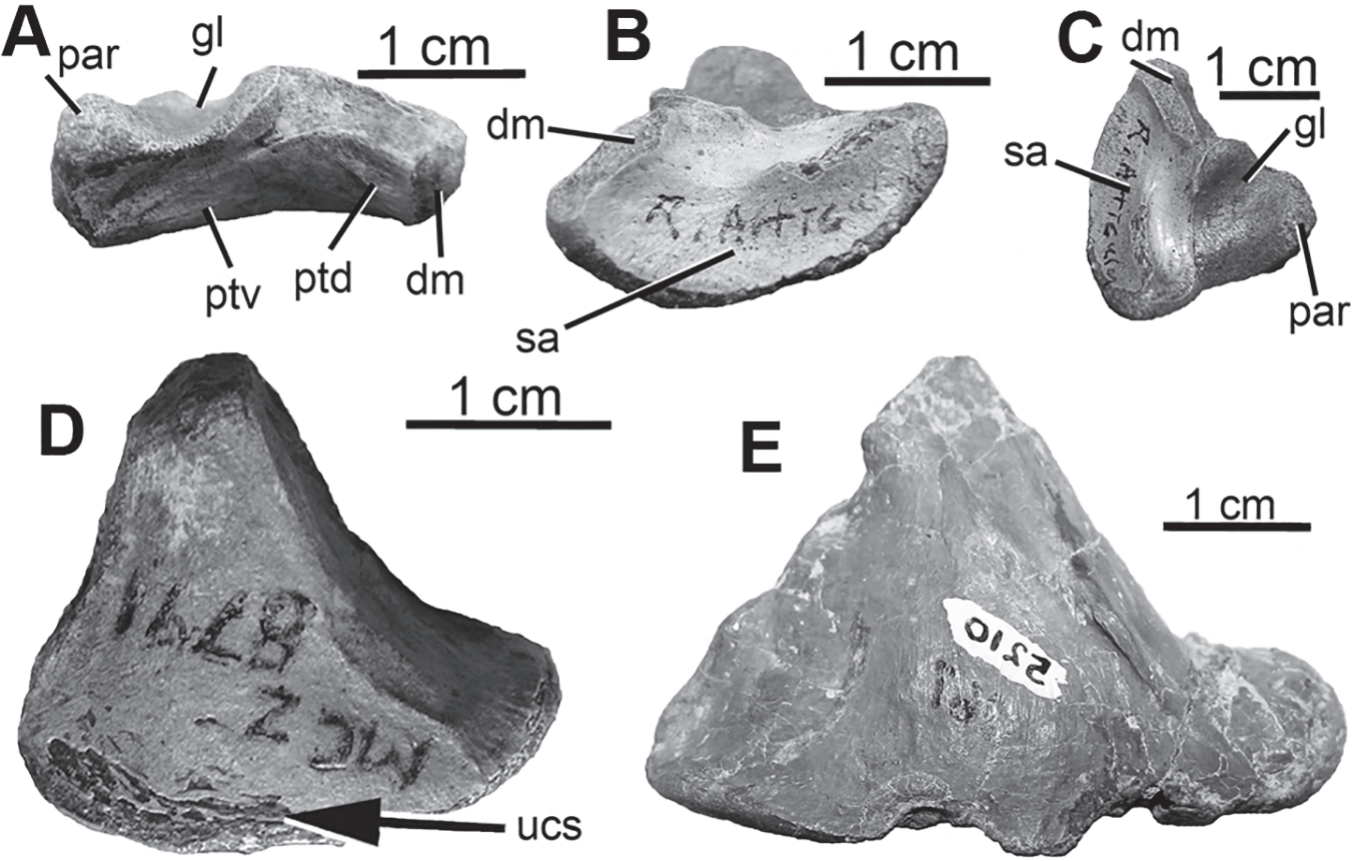
Two articulars of *D*. *antirrhopus*. **A**, medial, right articular, MCZ 8791; **B**, lateral, right articular, MCZ 8791; **C**, dorsal, right articular, MCZ 8791; **D**, ventral, right articular, MCZ 8791; **E**, image horizontally flipped, ventral, left articular, YPM 5210. **Abbreviations**: **dm**, area of insertion of M. depressor mandibulae; **gl**, glenoid; **par**, area of contact with prearticular; **ptd**, probable insertion point of M. pterygoideus dorsalis; **ptv**, probable insertion point of M. pterygoideus ventralis; **sa**, area overlain by the surangular; **ucs**, undeveloped cortical surface.

**Fig 6:**
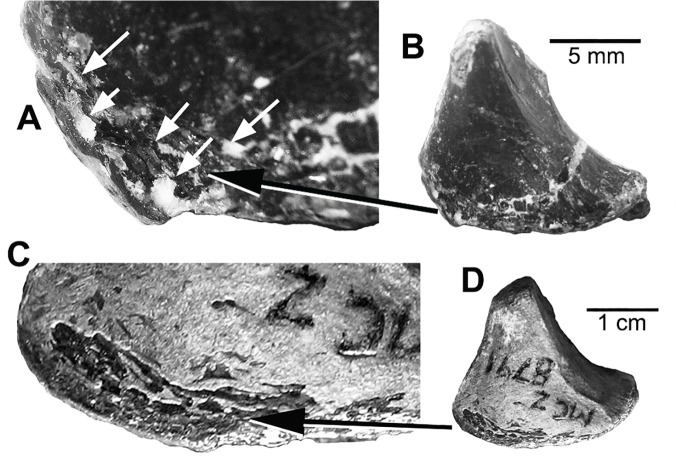
Articular foramina. **A**, detail of right articular foramina in *B*. *feinbergorum*, AMNH FR 30554; **B**, ventral view of right articular of *B*. *feinbergorum*, AMNH FR 30554; **C**, detail of right articular foramina in *D*. *antirrhopus* MCZ 8791; **D**, ventral view of right articular of *D*. *antirrhopus* MCZ 8791.

The ratio created from our standard measurement of the length of the articular and the measurement from the area of insertion for the M. depressor mandibular to the lateral end of the area overlain by the surangular in MCZ 8791 is. 62 and 1.05 in YPM 5232. In the character of the measurement from the area of insertion for the M. depressor mandibular to the lateral end of the area overlain by the surangular, the juvenile condition in MCZ 8791 is shorter than our standard length of the articular; in contrast, in the adult condition in the YPM 5232 articular, this same distance is longer. As in the distance between the caudal edge of the promaxillary fenestra and the rostral edge of the antorbital fenestra in the juvenile MCZ 8791 maxilla, these ratios in the articular indicate that the lower jaw in MCZ 8791 as well as the maxilla were relatively shorter than in the adult condition.

The ratio created from the dorsoventral height to our standard measurement of the length of the articular in MCZ 8791 is. 44 and. 80 in YPM 5232. In the character of the dorsoventral height of the articular, the juvenile condition in MCZ 8791 is shallower in contrast to the height that is the adult condition in YPM 5232. This more gracile dorsoventral morphology is similar to the narrower, juvenile, mandibular morphology of Carr’s Stage 1 specimens of members of Tyrannosauridae [33,34,35].

In the ventral view of the MCZ 8791 articular, the juvenile condition exhibits the character of a complex of small foramina separated by thin, trabecular-like bone struts and surrounded by thin, developing cortical bone (Fig 6); in contrast, this complex of foramina is not present in the adult condition as in YPM 5210 (Fi. 5E). In the juvenile articular the trabeculae are emerging from within the matrix that in-fills the articular. This indicates that the internal composition of this articular is not hollow or pneumatic but filled with an “interlacing” complex of trabeculae.

#### Dentition

MCZ 8791 preserves one complete maxillary tooth (Fig 4A and 4C). The mesial edge of that tooth is located directly below the ventral end of the promaxillary fenestra. Its basal mesial/distal width is 40% that of the corresponding tooth in the YPM 5232 left maxilla. In the character of the gaps between alveoli, although slightly variable in length, in the juvenile MCZ 8791 maxilla the gaps are comparatively wider than in the adult condition exemplified by the YPM 5232 left maxilla.

The juvenile condition of the angle of ventral-distal tooth orientation on MCZ 8791 is 52° (Fig 4C) which is less acute than the same angle of the maxillary teeth on the adult YPM 5232 at 46° (Fig 4D). The maxillary bone immediately surrounding the base of the YPM maxillary teeth shows no evidence of taphonomic distortion effecting this orientation. In an adult *D*. *antirrhopus* the apical angle of ventral-distal orientation of the individual denticles on the distal side of a maxillary tooth is approximately 50° to the long axis of the tooth. When the long axis of these distal denticles are realigned due to the ventral-distal “raking” angle of the maxillary teeth, the denticles become oriented parallel to the ventral edge of the maxilla. The character of this parallel orientation is an adult condition.

### Description: postcranial anatomy of MCZ 8791

#### Cervical vertebra

**Fig 7:**
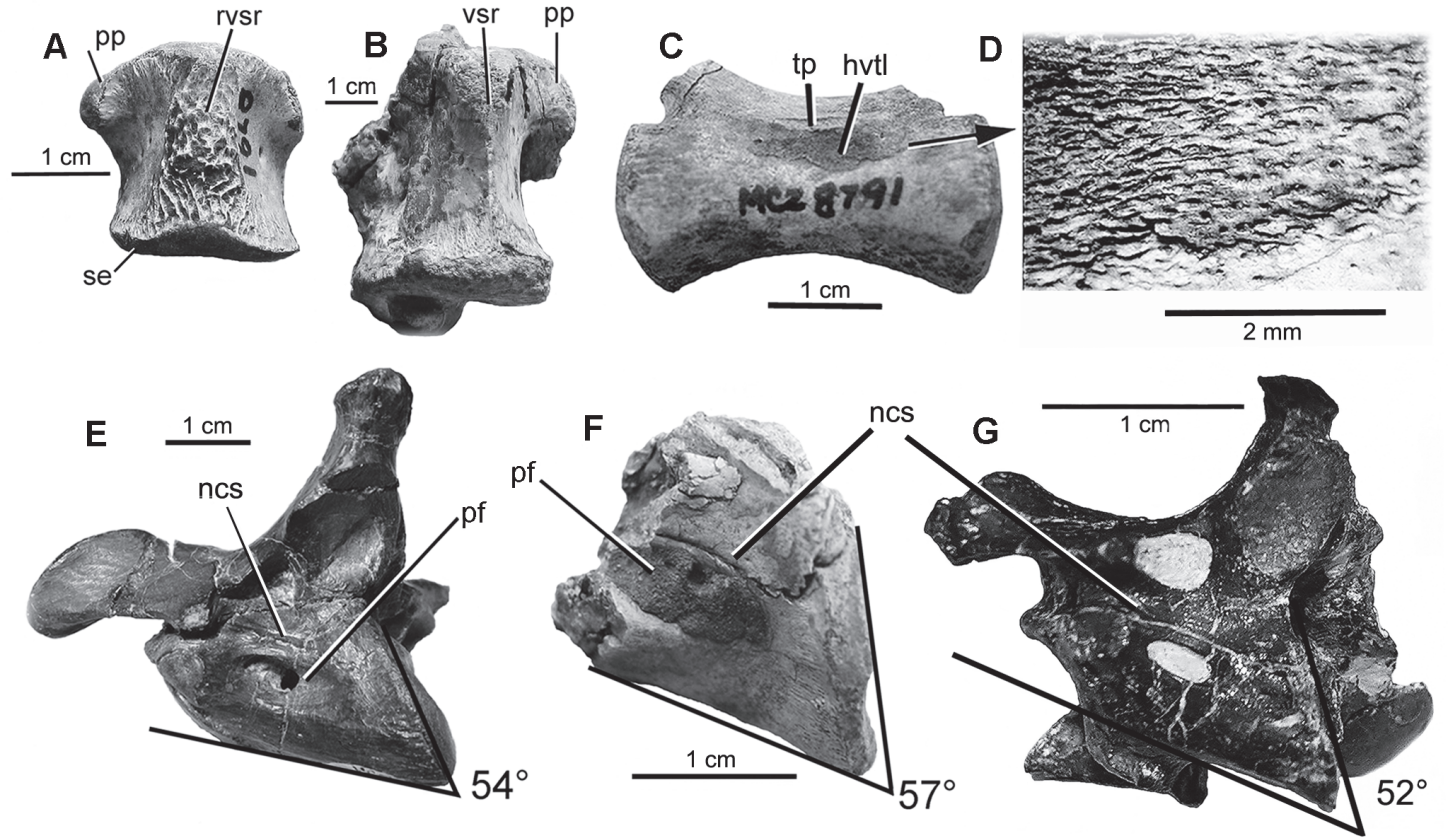
Vertebrae of *D*. *antirrhopus*. **A**, rugose, ventral sagittal ridge on dorsal vertebra of MCZ 8791; **B**, smooth ventral sagittal ridge on dorsal vertebra of MOR 1178. **C**, lateral view of mid-caudal vertebra, MCZ 8791; **D**, highly vascularized tissue layer on top of cortical bone of mid-caudal vertebra, MCZ 8791; **E**, seventh (?) cervical vertebra, YPM 5210; **F**, fragmentary seventh (?) cervical vertebral centrum, MCZ 8791; **G**, third (?) cervical vertebra, *B*. *feinbergorum*, AMNH FR 30554. **Abbreviations**: **hvtl**, highly vascularized tissue layer; **pf**, pneumatic foramen; **pp**, parapophysis; **rvsr**, rugose ventral sagittal ridge; **tp**, transverse process; **vsr**, ventral sagittal ridge; **se**, sharp edge on the posterior articulating surface.

One cervical vertebra was recovered (Fig 7F). It is a posterior portion of a centrum. The 57° oblique angle of the circular, platycoelous, articular surface most closely matches the 54° angle of orientation on the seventh (?) cervical vertebra of YPM 5210 [1].

In the MCZ 8791 cervical vertebra, the neurocentral suture lies immediately above the long groove-like lateral pneumatic fossa. That is the juvenile condition. This suture is more dorsally situated in YPM 5210, which is the adult condition (Fig 7E).

As determined by Brochu (1996), the fusion of neurocentral sutures in the cervical vertebrae of crocodilians is consistent with morphological maturity [36]. The neurocentral suture is open in the MCZ 8791 cervical vertebra, which is the juvenile condition. It is fused in the adult specimen YPM 5210.

The lateral profile of the caudal margin of the articulating surface of the centrum is convex in YPM 5210, which is the adult condition. It is slightly concave in MCZ 8791, which is the juvenile condition. In lateral view, the ventral profile of the centrum in the MCZ 8791 juvenile condition is concave. It is straight in the adult YPM 5210 vertebra.

#### Dorsal vertebrae

All the neurocentral sutures in MCZ 8791 are unfused, which is the juvenile condition; in contrast, all of the adult YPM dorsal vertebrae possessed fused neurocentral sutures. On the lateral surfaces of one of the four intact centra, there are small pneumatic foramina (horizontal width is 1.14 mm). In a mid-dorsal vertebra of MCZ 8791, the ratio between the horizontal length of the pneumatic foramen and the length of the centrum is. 05. In the fourth dorsal vertebrae of YPM 5204, the same ratio is. 58. In the character of the horizontal length of the pneumatic foramen on the lateral surface of the centrum of the dorsal vertebrae, the juvenile condition is only 9.2% the length on the adult YPM 5204 vertebra.

All the MCZ 8791 centra possess a complex, rugose, ventral sagittal ridge (Fig 7A). This is the juvenile condition. The morphology on the ventral portion of a similar dorsal vertebra of the adult specimen of *D*. *antirrhopus* MOR 1178 is smooth sided with a singular blade-like ventral sagittal ridge (Fig 7B). This is the adult condition.

The articulating surfaces of the MCZ 8791 centra possess sharp circumferential edges (Fig 7A). This is the juvenile condition. On the adult MOR 1178 dorsal vertebra (Fig 7B) these same edges are more rounded.

Open minute foramina on the subtly textured, articulating surfaces of the centra is evidence of open, cartilaginous tubules and cartilaginous end caps which is the juvenile condition [37]. The adult condition is smooth without evidence of any open tubules. Similar open tubules have been found on juvenile ornithischian dinosaurs [7].

The development of the parapophysis on the MCZ 8791 dorsal vertebrae (Fig 7A) is disproportionately smaller than that on the MOR 1178 dorsal vertebra. The juvenile condition possesses no caudal expansion to the articulating surface as is the case on the adult MOR 1178 vertebra (Fig 7B). A rugose surface with minute foramina exists around the peripheral edges of the articular facet of the parapophysis in the juvenile specimen MCZ 8791. The adult condition of the parapophysis on the MOR 1178 vertebra does not possess these minute foramina.

#### Caudal vertebrae

One of the three proximal caudal, vertebral centra recovered possessed the largest vertebral dimensions (Table 1). The ratio of the height to the width of the proximal end of the centrum of this juvenile MCZ 8791 caudal vertebra is. 94. The same ratio in the adult YPM 5210 fifth proximal vertebra is. 90. The ratio of the height the proximal end of the centrum to the length of centrum in this juvenile MCZ 8791 caudal vertebra is. 82. The same ratio in the adult YPM 5210 fifth proximal vertebra is. 77. These ratios are evidence of a similarity in the morphology of these two vertebrae.

**Table 1:** Comparative measurements (mm) of vertebrae.

	YPM 5210	MCZ 8791		rat of YPM 5210
**width of anterior articulating surface of centrum (mm)**				
7^th^ (?) cervical	38.84		15.23	.39
11^th^ (?) dorsal	64.50		25.96	.40
5^th^ (?) prox caudal	38.48		15.17	.39
**height of anterior articulating surface of centrum (mm)**				
7^th^ (?) cervical	42.22		16.10	.38
11^th^ (?) dorsal	66.76		23.00	.34
5^th^ (?) prox caudal	34.48		14.32	.42
**rostrocaudal length of centrum (mm)**				
11^th^ (?) dorsal	44.76	17.38		.39

Abbreviations: mm, millimeter; prox, proximal; rat, ratio.

On a mid-caudal vertebra (Fig 7C and 7D), there is a fragment of a thin, highly vascularized, striated, periosteal bone layer overlying far less porous cortical bone. This layer may be a juvenile character in *D*. *antirrhopus*. It does not appear on any other examined MCZ, MOR, or YPM vertebrae of this taxon, but the simple lack of such an easily detachable feature is not strong proof of its ontogenetic absence.

This layer possesses a longitudinal series of small striations which is the juvenile condition. This differs from the smooth cortical surface on the mid-caudal vertebra of the adult specimen YPM 5203. Similar striations have been described on the cortical surfaces of juvenile bone of tyrannosaurids, pterosaurs, and ceratopsians [33,38,39]

#### Pectoral Girdle

The comparative ratios between the measurements of the right coracoid of MCZ 8791 and those of the YPM 5236 right coracoid are in Table 2. When compared to the YPM 5236 coracoid, the coracoids of MCZ 8791 and the coracoid of the immature *D*. *antirrhopus* specimen OMNH 50268 all possess the juvenile condition of a less caudodorsally flared, glenoid lip, as originally described by Brinkman et al. (1998) [4]. This is in contrast to the more strongly flared glenoid lip which is the adult condition in the YPM 5236 coracoid.

**Table 2:** Comparative measurements (mm) of shoulder girdle and forelimb elements.

**Coracoids**	**YPM 5236**	**OMNH 50268**	**lt MCZ 8791**	**rt. MCZ 8791**	**rat of YPM 5236**
med/lat scap glen sut	25.73	16.44	15.17	15.59	.61
dors/vent scap glen sut	29.95		21.97	21.84	.73
med/lat acrocora proc	20.45		12.25	11.97	.59
acrocora/glen length	29.20	22.20	19.47	19.97	.68
**lt radius prox end**	**AMNH 3015**	**YPM 5220**	**YPM 5230**	**MCZ 8791**	**rat of AMNH 3015**
prox trans. width	20.6*	20.2*	23.6	14.11	.68
least dia of shaft	10.0*	9.0*	9.3*	6.71	.67
**rt ulna**	**AMNH 3015**	**YPM 5220**	**YPM 5230**	**MCZ 8791**	**rat of YPM 5220**
prox trans width	31.2*	29.0*	34.1*	18.17	.63
dist end lst dia of shaft	11.8*	10.6*	13.2*	8.42	.79
dist end trans width	28.3*	30.0/*	35.5*	19.54	.65

**rt manual II-2 phal**	**AMNH 3015**	**YPM 5211**	**lt YPM 5206**	**YPM 5209**	**MCZ 8791**	**rat lt YPM 5206**
length	70.7*	76.5*	75.96	78.0*	56.22	. 74
dist trans width	12.9*	13.6*	13.61	13.6*	9.04	.66
prox trans width	13.7*	14.1	14.50	14.0*	9.05	.62
mid-shaft trans width			10.12	10.98	6.15	.61
dist end height			14.60	14.62	9.22	.63
prox end height			21.42	crushed	13.65	.64
mid-shaft height			10.32	9.81	6.52	.63

* Measurement taken from Ostrom [1].

Abbreviations: acrocora, acrocoracoidal process; dia, diameter; dist, distal; dors, dorsal; glen, glenoid; lat, lateral; lst, least; lt, left; med, medial; phal, phalanx; proc, process; rt, right; scap, scapula; sut, suture; trans, transverse; vent, ventral.

The coracoid of MCZ 8791 possesses a smooth, sutural, articular surface which is the juvenile condition. In the coracoid of the adult specimen YPM 5236, the condition is a surface possessing rugose digitation. Previously, this juvenile condition has been recognized in the sub-adult specimen OMNH 50268 [4,40].

When two ratios are created contrasting the measurement between the acrocoracoidal process and the edge of the glenoid and the mediolateral width of the coracoid/scapula suture at the glenoid, in the YPM 5236 the ratio is 1.13. In the right coracoid of MCZ 8791, the same ratio is 1.28. As to the character of the length of the coracoid, the juvenile condition of the length of the MCZ 8791 coracoid is comparatively longer than the adult condition in YPM 5236.

#### Ulna

**Fig 8:**
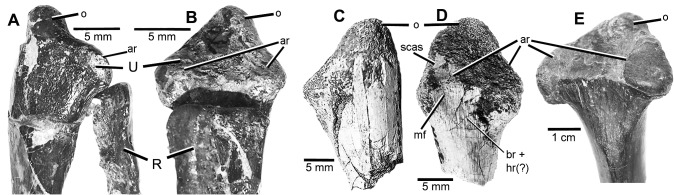
Proximal end of ulna. **A**, medial view of proximal end of left ulna and radius of *B*. *feinbergorum*, AMNH FR 30554; **B**, cranial view of proximal end of left ulna and radius of *B*. *feinbergorum*, AMNH FR 30554; **C**, lateral view of proximal end of right ulna of *D*. *antirrhopus*, MCZ 8791; **D**, cranial view of proximal end of right ulna of *D*. *antirrhopus*, MCZ 8791; **E**, cranial view of proximal end of adult *D*. *antirrhopus* ulna YPM 5230. **Abbreviations**: **ar**, articulating facets; **br + hr (?)**, possible insertion areas of the M. brachialis and M. humeroradialis from Ostrom [1]; **mf**, minute foramina; **o**, olecranon; **R**, radius; **scas**, smooth cortical articular surface; **U**, ulna.

The proximal end of the right ulna of MCZ 8791 possesses a low but distinct cranial/proximally-oriented olecranon process which is the juvenile condition (Fig 8C and 8D). Although Ostrom’s original description did not include an olecranon on the ulna, the triangular morphology of the proximal end of the left ulna of YPM 5230 is made up of the two articular facets and a diminished olecranon process (Fig 8E). In comparison to the olecranon process in MCZ 8791, the olecranon in YPM 5230 is flattened and proximally oriented to a plane similar to that of the two articular facets which is the adult condition (Fig 8E).

In cranial view (Fig 8D), on the proximal end of the right ulna in MCZ 8791, the dorsoventral dimension is the largest, and the articular facets are semi-cranially oriented. That is the juvenile condition. In cranial view (Fig 8E), the medial/lateral dimension of the proximal end of the adult left ulna, YPM 5230, is the largest, and the articular facets are proximally oriented which is the adult condition.

The proximal end of this juvenile ulna possesses minute foramina in the synovial region that are similar to those found on the articular surfaces of the vertebral centrum (Fig 8D) [39]. These tubules indicate further cartilaginous development of this proximal end of the ulna as is found in *Alligator mississippiensis* [41]. A small portion of a smooth, cortical, articular surface remains (Fig 8D). This smooth surface is a juvenile condition that contrasts to the finely textured, rugose surface described by Ostrom for the adult condition [1].

When two ratios are created, between the transverse width of the distal end and the transverse width of the proximal end of the ulna, the ratio in the YPM 5220 ulna is. 41. In MCZ 8791, the same ratio is. 56. As to the character of the transverse width of the distal end of the ulna in the juvenile MCZ 8791, it is relatively larger than the same width in the left ulna of the adult YPM 5220. In a direct comparison between the transverse width of the distal end of the ulna in MCZ 8791 and that of YPM 5220 the ratio is. 65.

#### Right manual II-2 phalanx

The right manual II-2 phalanx is the only complete forelimb bone recovered. The size and proportional differences between this bone and the adult specimen, YPM 5206 are in Tables 2 and 3. When two ratios are created, between the transverse width of the proximal end of the manual II-2 phalanx and the length, the ratio in AMNH 3015 is. 19. In MCZ 8791, the same ratio is. 16. As to the character of the length of the manual II-2 phalanx, the juvenile condition of the length of the MCZ 8791 manual II-2 phalanx is comparatively longer than the adult condition in AMNH 3015. When the length of the right, manual II-2 phalanx in MCZ 8791 is directly compared to that in the adult AMNH 3015 the result is a ratio of. 78. That same ratio in relation to the largest manual II-2 phalanx specimen YPM 5209 is.72. The ratios in Table 3 indicate that the juvenile condition of the MCZ 8791 phalanx was comparatively thinner and longer than that of the adult condition in the YPM 5206 phalanx.

**Table 3:** Ratios within right manual II-2 phalanges.

rt manual II-2	mdsft/lgth	dswth/lgth	prxwth/lgth
**YPM 5206**	.13	.18	.25
**MCZ 8791**	.12	.16	.17
**FR 30554**	.12		

Abbreviations: mdsft, midshaft transverse width; lgth, length; dswth, distal end width; prxwth, proximal width; rt man II-2, right manual II-2 phalanx.

#### Pelvic Girdle

The ilium and ischium were completely co-ossified by this early growth stage. Thus, the fusion of ilium and ischium is not a reliable adult character. This is similar to the completely fused, adult condition as described in the dromaeosaurid, *H*. *elizabethae* [11].

#### Femur

In comparison to the smooth perimedullary layer within the adult femur of MOR 1178, the MCZ 8791 juvenile condition of the inner surface of the medullary cavity has an irregular “sponge-like” appearance. In the juvenile MCZ 8791, the ratio between the thickness of the perimedullary layer (.38 mm) and the thickness of the cortical side of the bone (4.49 mm) is. 09. In the adult MOR 1178, the ratio between the thickness of the perimedullary layer (1.17 mm) and the thickness the cortical side of the bone (6.53 mm) is. 18. The juvenile perimedullary layer in MCZ 8791 is comparatively thinner than adult perimedullary layer in MOR 1178.

In MCZ 8791, the ratio between the thickness of the cortical side of the bone (4.49 mm) to the thickness of the distal condylar portion of the bone matrix (7.40 mm) is. 60. In MOR 1178, the ratio between the thickness of the cortical side of the bone (6.53 mm) to the thickness of the distal condylar portion of the bone matrix (12.24 mm) is. 53. The adult condition of the distal condylar bone matrix of the MOR 1178 femur is comparatively thicker than in the juvenile condition in MCZ 8791.

#### Left Fibula (Fig 9)

At the point of cross-sectional breakage in the shaft of the left fibula of MCZ 8791, the ratio of the cranial/caudal width of the medullary cavity (6.52 mm) to the cranial/caudal diameter of the entire cross section (10.87 mm) is. 60. This is the juvenile condition. In the MOR 1178 left fibula, the ratio of the cranial/caudal widths of the medullary cavity (6.06 mm) to the cranial/caudal diameter (13.12 mm) is. 46. This is the adult condition.

**Fig 9:**
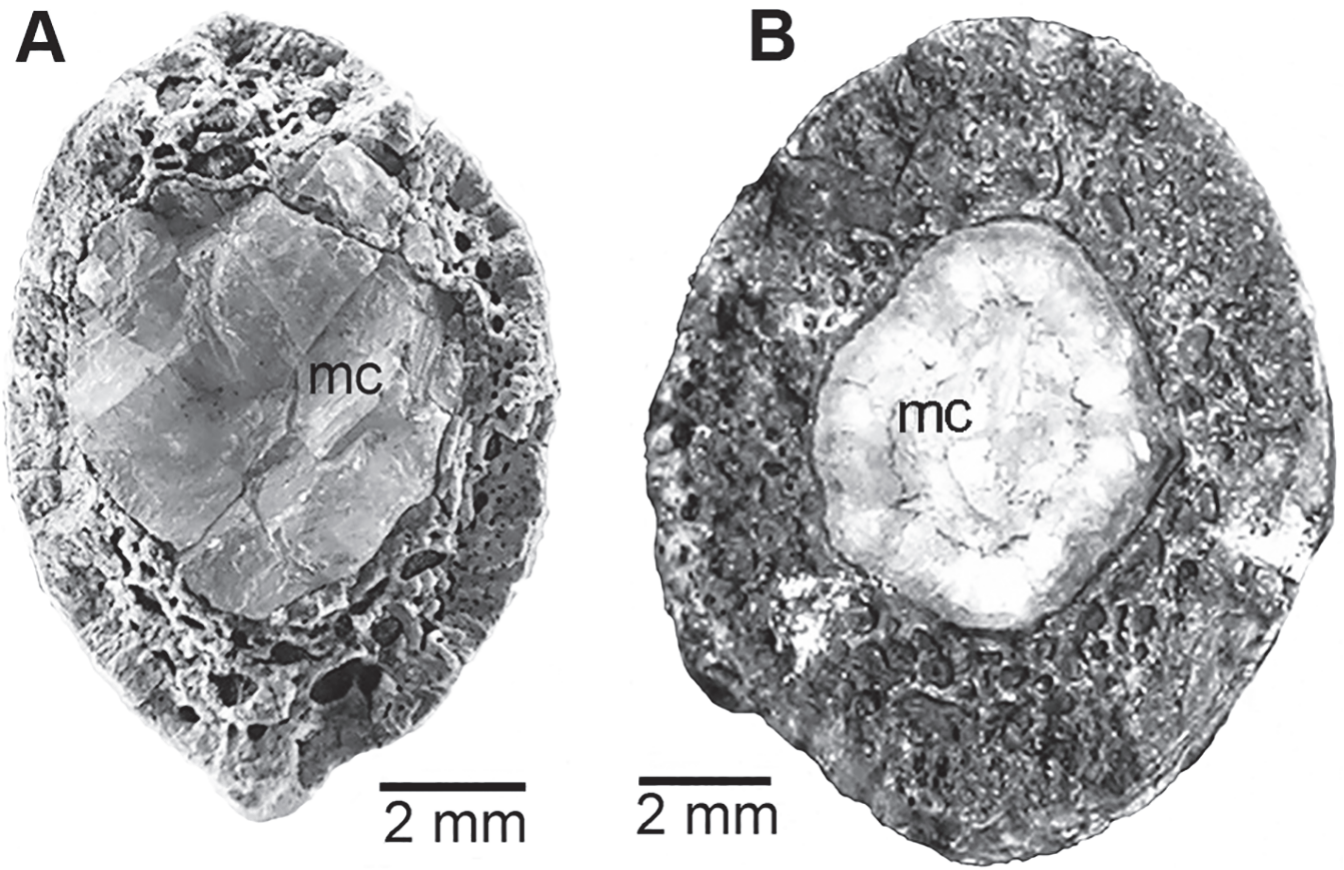
Mid-shaft cross-section of fibulae on *D*. *antirrhopus* specimens MCZ 8791 and MOR 1178. **A**, mid-shaft cross-section, MCZ 8791; **B**, mid-shaft cross-section, MOR 1178.

In MCZ 8791, the ratio of the mediolateral widths of the medullary cavity (5.20 mm) to the mediolateral diameter of the entire cross section (7.37 mm) is. 71. This is the juvenile condition. In MOR 1178 at the point of breakage, the ratio of the mediolateral widths of the medullary cavity (5.77 mm) to the mediolateral diameter of the entire cross section (10.59 mm) is. 55 which is the adult condition. The juvenile condition of the matrix in the MCZ 8791 fibula (Fig 9A) is comparatively thinner in relation to the size of the medullary cavity than in the adult MOR 1178 fibula (Fig 9B). Other measurements of the fibulae are in Table 4.

**Table 4:** Comparative measurements (mm) of hind limb elements.

**rt tibia**	**AMNH 3015**	**MCZ 8791**	**rat of AMNH 3015**			
prox width	44.8*	31.51	.70			
max prox dimension	74.0*	41.27	.56			
distal med/lat width	63.3*	40.67	.64			
**lt fibula prox end**						
prox med/lat width	16.00*	8.25	.52			
prox cra/cau width	48.40*	39.00	.81			
**rt ped II-3**	**AMNH 3015**	**YPM 5205**	**YPM 5218**	**MCZ 8791**	**rat of YPM 5205**	
facet height	27.5*	35.0*	31.0*	20.17	.58	
prox trans width	11.9*	12.4*	11.9*	7.56	.61	
flex tub below facet		7.22	4.25	2.63	.36	
max dors/vent height		40.97	37.17	23.68	.58	
**rt ped II-1 prox**	**lt AMNH 3015**	**lt YPM 5205**	**YPM 5207**	**lt YPM 5217**	**MCZ 8791**	**rat of YPM 5205**
prox trans width	25.2*	23.0*	22.2*	21.6*	15.5	.67
prox height		24.25		25.10	15.56	.64
mid-shaft height		13.40		11.45	11.64	.87
**lt ped IV-1 prox**	**AMNH 3015**	**rt YPM 5205**	**YPM 5207**	**YPM 5217**	**MCZ 8791**	**rat of AMNH 3015**
prox trans width	19.9*	22.3*	21.5*	18.1*	11.98	.60
**rt ped IV-4**	**lt AMNH 3015**	**AMNH 3015**	**YPM 5205**	**lt YPM 5205**	**MCZ 8791**	**rat of AMNH 3015**
length	26.3*	25.0*	30.24*	28.65*	14.47	.58
				rt. YPM 5207		
prox trans width	14.2*	14.0*	15.2*	16.0*	10.59	.76
						**rat of YPM 5205**
prox height			16.88	15.94	10.79	.64

*Measurements from Ostrom [1].

Abbreviations: cra/cau, cranial/caudal; dors/vent, dorsal/ventral; flex tub, flexor tubercle; max, maximum; med/lat, medial/lateral; ped, pedal phalanx/ungual.

#### Tibia

**Fig 10:**
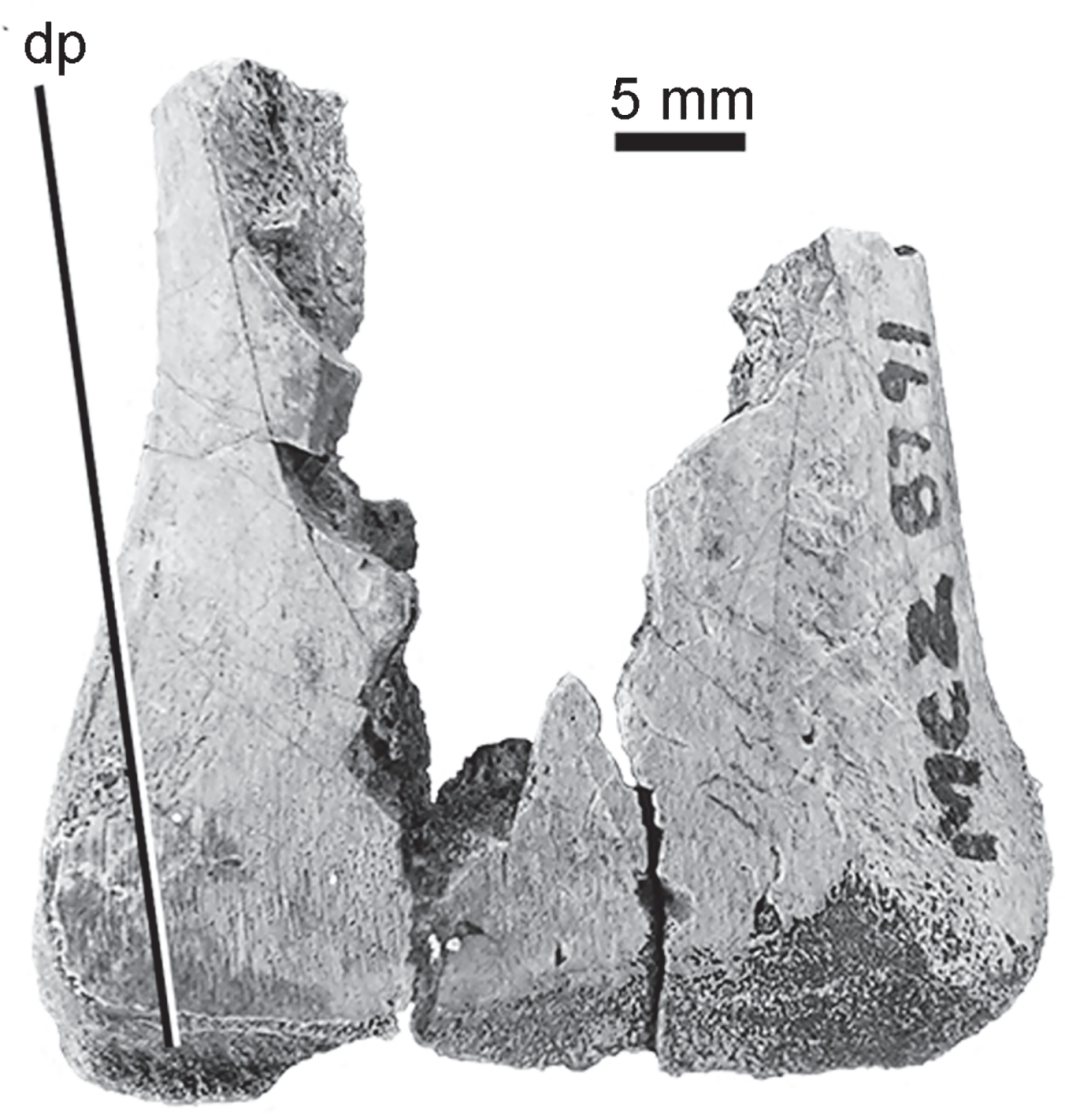
Descending process on the distal end of the tibia in *D*. *antirrhopus*. **Abbreviation**: **dp**, descending process of the tibia.

In the MCZ 8791 tibia, the proximal and distal end is covered with numerous minute foramina, which is the juvenile condition. These foramina are not present in the adult condition of the proximal end of the tibia of MOR 1178. A new feature in *D*. *antirrhopus* discovered in MCZ 8791 is a descending process that lies in front of the position of the medial portion of the astragalus (Fig 10). In AMNH 3015, this tibial descending process is obscured by the articulating calcaneum and astragalus.

#### Pedal II-1 phalanx

When two ratios are created, between mid-shaft height and the transverse width of the proximal end of the pedal II-1 phalanx, the ratio in the right YPM 5205 pedal II-1 phalanx is. 58. This is the adult condition. In the juvenile MCZ 8791, the same ratio is. 75. As to the character of the mid-shaft height of the pedal II-1 phalanx, in the juvenile MCZ 8791 it is relatively larger than the same height on the pedal II-1 phalanx in YPM 5205. The result of a direct comparison between the mid-shaft heights of the MCZ 8791 pedal II-1 phalanx and that of YPM 5205 is a ratio of. 87.

#### Pedal II-3 ungual (Fig 2)

In the juvenile MCZ 8791, the ratio between the distance from the apex of the flexor tubercle to the ventral limit of the proximal articulating facet and the height of the proximal articulating facet is. 25. In adult specimen YPM 5205, the same ratio is. 30. These ratios indicate that the character of the distance from the flexor tubercle to the ventral limit of the proximal articulating facet in the juvenile specimen MCZ 8791 is comparatively shorter in contrast to the adult condition in the holotype. This ratio in the second pedal ungual of MCZ 8791 is similar to the. 23 ratio in the sub-adult specimen OMNH 50268 (Fig 2E). As in MCZ 8791 and in OMNH 50268, this smaller distance between the flexor tubercle to the ventral limit of the proximal articulating facet is due to the juvenile condition of the flexor tubercle being comparatively smaller than in the adult YPM 5205.

### Comparisons to *B*. *feinbergorum* (AMNH FR 30554)

If the pattern of the ontogenetic transformation of these informative characters is conservative within other members of Dromaeosauridae [42], then at least some of the characters within this pattern could be diagnostic of the age of individuals within other dromaeosaurid taxa. Osteological comparisons have been made to *B*. *feinbergorum* (AMNH FR 30554), a known sub-adult of the taxon [8,9]. AMNH FR 30554 had reached 75% the size of an adult of that taxon [8]. *B*. *feinbergorum* exhibits an extremely long forelimb morphology that is similar to the forelimb bones in the juvenile *D*. *antirrhopus* specimen MCZ 8791. Without a recognized juvenile and adult specimen of *B*. *feinbergorum* to bracket the ontogenetic variability in that taxon, the specific growth stage of the sub-adult specimen AMNH FR 30554 cannot be determined, although its skeletal morphology is similar to what would be expected in a specimen from a transitional growth stage between juveniles and adult in *D*. *antirrhopus*. Observing the presence of some of these juvenile morphological characters is a first step in determining a possible shared conservative ontogenetic pattern within this second taxon.

#### Angle of orientation of the maxillary teeth and alveolar gap

The angle of the ventral-distal orientation of the long axis of the maxillary teeth of *B*. *feinbergorum* is 40°. This angle is even more acute than that found in the mature YPM 5232 left maxillary dentition (Fig 4D). This more acute ventral-distal tooth orientation is an adult condition in *D*. *antirrhopus*. Also, the gap between the maxillary alveoli is intermediate in relative size between the juvenile and adult condition in *D*. *antirrhopus*.

#### Morphology of maxillary fenestra

*B*. *feinbergorum* does possess a relatively large maxillary fenestra. The horizontally elongate morphology of this fenestra is similar to that on the adult maxilla of YPM 5232 (Fig 4D). This is an adult condition in *D*. *antirrhopus*.

#### Articular cortical surface

There is an open area of foramina and undeveloped cortical bone on the surface of the articular. Any internal matrix cannot be observed. This morphology is similar to the same region on the articular of MCZ 8791 (Fig 6A and 6B). This is a juvenile condition in *D*. *antirrhopus*.

#### Cervical vertebrae

One cervical vertebra of *B*. *feinbergorum* (AMNH FR 30554) (Fig 7G) is similar to the seventh (?) cervical vertebrae of *D*. *antirrhopus*, YPM 5210 [1] (Fig 7E). For identification purposes, the 52° oblique angle of the caudal articular surface closely matches the 54° angle of the caudal articular surface on the seventh (?) cervical vertebra of YPM 5210 [1].

In AMNH FR 30554, the neurocentral suture lies farther above the long groove-like lateral pneumatic fossa in a position relatively half way between the juvenile position in MCZ 8791 which is closer to the pneumatic fossa, and the adult, more dorsal position of this suture as in the YPM 5210 vertebra. The position of this suture in the AMNH FR 30554 cervical vertebrae is intermediate between the juvenile and adult conditions found in *D*. *antirrhopus*.

The neurocentral suture is open in AMNH FR 30554 which is a juvenile condition in *D*. *antirrhopus*. It is fused in the adult condition in specimen YPM 5210. The lateral profile of the caudal margin of the articulating surface of the centrum is concave in AMNH FR 30554, which is the adult condition in the *D*. *antirrhopus* specimen YPM 5210. In lateral profile, the ventral margin of the centrum in AMNH FR 30554 is concave. This is the juvenile condition in the *D*. *antirrhopus* specimen MCZ 8791.

#### Rugose, ventral sagittal ridge on the centrum

The ventral surfaces of the cervical and/or dorsal vertebral centra on *B*. *feinbergorum* are either smoothly rounded or smooth sided, with simple keel-like sagittal ridges. They do not possess any of the complex rugose, ventral, sagittal ridges described on the dorsal vertebral centra on MCZ 8791. In *D*. *antirrhopus*, the smooth nature of these ventral surfaces and simple keel-like sagittal ridges is the adult condition.

#### Degree of sutural digitation on coracoid

Both the coracoid and scapula were recovered with AMNH FR 30554. Burnham et al. [8,9] note that the coracoid and scapula were unfused. The sutural articulating surfaces are smooth with no evidence of digitation. This is a juvenile condition in *D*. *antirrhopus* (4,40).

#### Ulna

The ulna of *B*. *feinbergorum*, AMNH FR 30554 (Fig 8A and 8B), possess a distinct olecranon process with a cranial/proximal orientation, which is the juvenile condition in *D*. *antirrhopus*. The left ulna in AMNH FR 30554 possesses two articular facets on the proximal end of the ulna as well as a developed olecranon. This differs from the published description of the proximal end of the right ulna, the morphology of which may be pathological in origin [9]. The olecranon of MCZ 8791 is similar to that in AMNH FR 30554.

#### Right manual II-2 phalanx

The right manual II-2 phalanx of *B*. *feinbergorum* (AMNH FR 30554) possesses some proportional similarities to that on MCZ 8791. The ratio between the midshaft width to the length of this phalanx is. 12 (Table 3). This is the same as the. 12 ratio in the MCZ 8791 manual II-2 phalanx, which is the juvenile condition in *D*. *antirrhopus*.

#### Fibula

The fibula of *B*. *feinbergorum* in AMNH FR 30554 is disproportionally thinner and more gracile than any of the fibulae of the specimens of *D*. *antirrhopus* (Fig 9). The fibula of *B*. *feinbergorum* possesses a relatively large medullary cavity and thin cortical walls. This is similar to the juvenile condition in MCZ 8791, but the differences in the morphology of the fibulae from these two taxa do not make accurate ontogenetic comparisons possible.

#### Open cartilaginous tubules

No open cartilaginous tubules can be observed on the articular surfaces of the available vertebrae on AMNH FR 30554, which is an adult condition in *D*. *antirrhopus*. Some are present upon the distal end of the ulna, the proximal end of the tibia, and the ends of the radius, but they are not as numerous as seen on MCZ 8791 limb bones. These open tubules are not present on the bones of any examined adult specimens of *D*. *antirrhopus*.

#### Conservative pattern in dromaeosaurid ontogeny

*B*. *feinbergorum* AMNH FR 30554 possesses six juvenile character conditions, four adult character conditions, and three intermediate character conditions, that are similar to the conditions in *D*. *antirrhopus*. When compared to these ontogenetic characters of *D*. *antirrhopus*, *B*. *feinbergorum* AMNH FR 30554 is probably more mature than MCZ 8791 but still transitional between the juvenile and adult ontogenetic pattern of characters; this would be consistent with the previous sub-adult age estimate for this specimen [8,9]. This comparative study proves that *B*. *feinbergorum* and *D*. *antirrhopus* share a morphologically similar ontogenetic pattern. This pattern was most probably inherited from a common ancestor. Given the close cladistic placement within Dromaeosauridae (Fig 1), we would expect *Saurornitholestes langstoni* [43] to share a similar ontogenetic pattern as well.

## Discussion

### Chronological age assessment

Between the juvenile and adult growth stages, certain characters and features are diagnostic of the chronological age that equates to these growth stages. Within a potential, complete growth series of this taxon, many of the juvenile size-independent or proportionately distinct characters previously described in this text should help to designate the specific growth stage of an individual specimen. One of the best, informative, variable characters that have been confirmed in other taxa [22] is the presence of minute foramina that are the open ends of cartilaginous tubules on the articulating surfaces of vertebral centra. Some of the other characters and/or features of similar importance would be the gracile morphology of the lower jaw and the alveolar region of the maxilla; the morphology of the maxillary fenestra; the presence of interdental plates; the ventral-distal orientation of the maxillary teeth; the open complex of foramina in the articular; the morphology of the cervical vertebrae; the complex, rugose, ventral sagittal ridge on the dorsal vertebrae; the thin periosteal layer on the mid-caudal vertebrae; the degree of digitation in the sutural surfaces of the scapulocoracoid suture; the morphology of the proximal end of the ulna; the histology of the radius; the inner surface and thickness of the perimedullary layer within the femur; the thickness of the distal condylar bone of the femur; the thickness of the cortical bone matrix of the fibula; the mid-shaft height in the pedal II-1 phalanx; and the size of the flexor tubercle on the pedal second ungual. Further discoveries of intermediate growth stage specimens would help to determine the rate and/or sequence within which these juvenile characters were transformed into their adult manifestations. Once such ontogenetic characters within a complete growth series have been defined, they would help to further differentiate the various taxa within Dromaeosauridae.

### Skeletal reconstruction (Fig 11)

**Fig 11:**
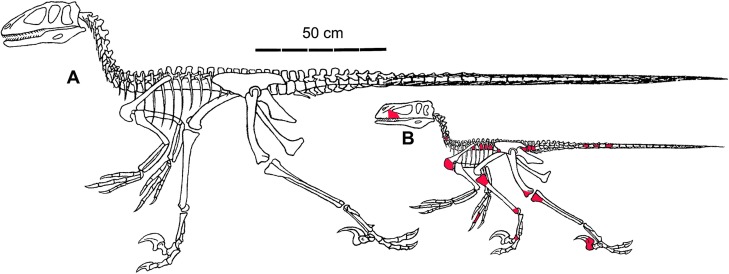
Skeletal reconstructions. **A**. Composite adult skeleton modified from Ostrom [1]. **B**. Reconstruction of MCZ 8791. Red tinting on bones illustrates the identification and distribution of the preserved, fragmentary bones of MCZ 8791. The positions of the thoracic and caudal vertebrae are approximated. Drawing of MCZ 8791 William Parsons 2014.

The overall cranial, trunk, and limb proportions of MCZ 8791 have been approximated from the available data in the tables and compared to the adult AMNH and YPM specimens. When compared to the adults, there are several proportional differences in MCZ 8791 (Fig 11). The cranial length is at 66.7% of the YPM adult cranial material; the dorsoventral height of the lower jaw is 41%. The comparisons to the YPM vertebrae were used to determine the trunk proportions. The length of the trunk is approximately 40% that of the YPM specimens. In comparison between the MCZ 8791 coracoids and the YPM 5236 coracoid, the dorsoventral length of the scapular glenoid suture is 73%, and the distance between the acrocoracoidal process and the glenoid is 68%. These proportions give an approximated size of 70% for the scapulocoracoid complex. The approximated length of the radius and ulna is 65%. The distal end of the ulna is 65% that of AMNH 3015. The manual bones are between 73% and 78% with the length of the MCZ 8791 manual II-2 phalanx at 78% that of the same phalanx in AMNH 3015. With the exception of the pedal II-3 ungual at 71%, the rest of the hind limbs are approximately 64% (Table 4). With some consideration for the position of the cervical vertebrae, the approximate body length of MCZ 8791 is 133.5 cm. This is compared to the length of the YPM/AMNH reconstruction which is in “normal posture” approximately 2.5 meters [1]. Beyond just proportional differences, the thin bone matrix, the presence of cartilaginous regions, and the long limb morphology of this juvenile specimen create a more gracile skeleton than that of the YPM specimens. This reconstruction bears some resemblance to the skeleton of the sub-adult specimen *B*. *feinbergorum*, AMNH FR: 30556, especially in regard to the length of the bones in the manual region of the forelimb [8,9].

In each of these two skeletal reconstructions (Fig 11) the pubis has been reoriented to a position sub-parallel to the tail. This is closer to the retroversion displayed in *V*. *mongoliensis* specimen IGM 100/985 [44], in *B*. *bondoc* [14], and the actual taphonomic position for the orientation of the pubis described for the *D*. *antirrhopus* specimen MCZ 4371 [45]. Given the greater length of the pubis compared to the ischium, the posterior extension of the pubic boot could have functioned as a further insertion surface for the M. ilio-ischiocaudalis.

### Body mass estimate

Without a complete cross-section of the femur, the body mass estimate is less precise than might have otherwise been calculated. Still, given the relationship between body mass and potential dromaeosaurid modes of mobility, there is some value in even an approximate estimate. To this end, working from the skeletal reconstructions, clay models were created. The clay model body mass estimate was cross-checked by lying a grid matrix of 1 mm squares across a large image (34 cm) of the two lateral, two-dimensional, illustrated bodies of the modified YPM/AMNH 3015 reconstruction and that of MCZ 8791 (Fig 11). The two-dimensional surface areas were then cubed using the centra width averages of the proximal, caudal vertebrae for these two specimens. Working from both John Ostrom’s [1] and Greg Paul’s [46] body mass estimates, the ratios between the two cubed body mass reconstructions were used to cross-evaluate the initial clay model estimated range for the body mass of MCZ 8791. Ostrom’s live weight estimate for the YPM reconstruction was 60 to 75 kg [1]. Greg Paul’s body estimate was 60 kg [46]. The live weight estimate for MCZ 8791 is between 6.95 to 8.70 kg.

### Potential for flight in juvenile *D*. *antirrhopus*

Within the literature, wing flapping as a form of dromaeosaurid/avian mobility has been suggested in relation to *Sinornithosaurus millenii* [47], and powered flight in other dromaeosaurids has been alluded to in discussions concerning aerodynamic dromaeosaurid feathers. Although the feathered forelimbs of some smaller dromaeosaurids possess some resemblance to avian wings, there are also some limitations to the ability to “flap” a dromaeosaurid wing in a manner similar to the supination/pronation movements of extant avians. One of the greatest limitations is the inability to rapidly rotate the humerus in a manner similar to what is found in most extant avians, as well as in the Enantiornithines [48]. The absence of a triosseal canal, the curvature of the shaft of the humerus, and the less well-developed, caudally projecting internal tuberosity on the head of the humerus [49], as is exemplified in *B*. *feinbergorum* and *D*. *antirrhopus*, would have inhibited any rapid rotation of the humerus.

Further, a differentiation of the M. scapulotricepitis tendon from the M. humerotriceps tendon is evidenced by their respective sulci on the distal end of the humerus in some Enanitiornithines as well as modern avians. These smaller muscles may help in stabilizing the wing in gliding [48] and help to transfer and/or control the momentum of the humeral rotation down the forelimb. These separate sulci are not found on the distal end of the humerus of *B*. *feinbergorum* or *D*. *antirrhopus*.

Still, the shallow, open morphology of the scapular glenoid in *D*. *antirrhopus* as well as its lateral orientation [3] would have allowed for a considerable arc of movement that was well within the range of functional avian wing flapping. This scapular glenoid would have allowed for the forelimb/wing to have been raised and lowered in a vertical manner and extended to some degree in a cranial direction. When those movements were combined with the contraction and the full rotation of the wrist, the extremely long manual elements, and the manual feathers, in a 190° lateral, rotational arc (which is the potential arc of rotation of the combined semi-lunate carpal and proximal end of the first metacarpal [1]), it would have greatly aided in the efficiency of dromaeosaurid wing flapping. Along with the contraction and expansion of the elbow joint while raising and lowering the forelimb, this rotation of the manual region of the dromaeosaurid forelimb would have created a primitive flapping movement that was comparable to the more efficient supination/pronation movements of the entire wing in extant avians.

When compared to the left ulna of YPM 5230, the more prominent and semi-cranial orientation of the olecranon process on the right ulna of MCZ 8791 indicates that the forelimb of the juvenile could have been extended farther than in the adult. The forelimb could have been extended to the point at which the straightened “locking out” position between the olecranon process of the ulna and the humerus was achieved. The diminished and re-oriented adult olecranon process would not be capable of articulating with the humerus without hyperextending the lower forelimb. This indicates a more permanently flexed position for the elbow joint in the adult [50]. The ontogenetic transformation of the olecranon process is evidence of a functional difference between the forelimbs of the juvenile and those of the adult.

The reasonably large distal end of the ulna in MCZ 8791 indicates that the wrist bones articulating with it were already substantial in size. This implies the early development of the asymmetric mobility potential for the wrist. This asymmetric mobility is an important milestone in the evolutionary development of avian flight [2,51].

Certain morphological features of some juvenile theropods, such as the longer legs of juvenile tyrannosaurids, have been interpreted as evidence of specifically enhanced juvenile behaviors [52]. Given the size of the bones of the wrist joint, the extreme length the manual bones, the enlarged scapulocoracoid complex, the overall exaggerated length of the forelimbs within the specimen MCZ 8791, the gracile trunk morphology, as well as the known flight potential possessed by other smaller feathered volant members of Dromaeosauridae, it is reasonable to speculate that the early exaggerated development of these juvenile features of the forelimb in *D*. *antirrhopus* would have enhanced juvenile flight behavior. This would have given these juvenile dromaeosaurids a mobility advantage similar to that which longer legs may have imparted to juvenile tyrannosaurids. In particular, the important function of the manual region of the juvenile dromaeosaurid forelimb in the process of primitive wing flapping would have gained a certain advantage by the enlarged development of this portion of the forelimb at an early growth stage within these taxa. This also explains the function of the extremely long bones in manual region of the forelimbs of the sub-adult specimen of *B*. *feinbergorum*. All of this would create in MCZ 8791 a potential for flight comparable to that of other smaller potentially volant dromaeosaurids such as *S*. *millenii* (IVPP V 12811) [47], *M*. *gui*, IVPP V13352 [53], *M*. *zhaoianus*, IVPP V 12330 [54], and *C*. *yangi*, HG B016 [55].

In particular, the morphology of *C*. *yangi* suggests that aerial locomotion was not limited to small-bodied dromaeosaurids but was also present among more sizable members of Dromaeosauridae [55]. The body mass estimate for *C*. *Yangi*, HG B016, is 4.0 kg [55]. The biodynamics that are necessary for dromaeosaurid flight as exhibited by *C*. *yangi* make speculating as to the flight capability of MCZ 8791well within the realm of reason.

As to the possible limitation of forms of avian flight due to body mass, the weight limit for extant volant birds is considered to be around the size of the Kori Bustard (*Ardeotis kori*), at about 18 kg [56]. There were extinct avian taxa which were exceptions to the weight limitation of extant volant birds, such as *Argentavis magnificens* [57,58] and some members of Pelagornithidae, such as *Pelagornis chilensis*, MNHN SGO.PV 1061 [59]. The estimated weight of MCZ 8791 is well below the weight/size limitation exhibited by both extinct and extant volant birds. The live weight of MCZ 8791 would not have been a limiting factor to flight potential.

Given the increase in body mass/weight, the change in the morphology of the olecranon process, and the reduction in relative forelimb length between this juvenile, MCZ 8791, and the more mature specimens within *D*. *antirrhopus*, such volant behavior may have been limited to only the earlier growth stages of this taxon. Within extant nonvolant avian taxa, there are examples of the successful flight capability of juveniles such as those of the flightless rail [60] and Giant coot (*Fulica gigantea*) [61]. These examples set a precedent for such an ontogenetic loss of flight.

## Conclusion

Our primary findings are as follows. The comparative ratios between the dimensions of the juvenile vertebrae of MCZ 8791 and adult YPM 5210 range between. 34 and. 42 (Table 1). The dimensions and ratios found within the skull, forelimb bones, and hind limb bones of the juvenile *D*. *antirrhopus* specimen MCZ 8791 indicate that the juvenile skull and limb bones are larger in comparison to the vertebral column in MCZ 8791 than in adult specimens of *D*. *antirrhopus*. In addition, the juvenile maxilla and lower jaw are relatively shorter in length and shallower in height than the adult condition in YPM 5232. The ratios within the coracoid and ulna indicate early growth stage enlargement of the skeletal and muscular morphology of the shoulder girdle and forelimb. Although fewer actual lengths have been determined for the hind limb bones, the ratios between the dimensions of the juvenile hind limb bones in MCZ 8791 and the same bones of adult specimens of *D*. *antirrhopus* range between. 36 and. 87. Similar to the forelimbs, this indicates that an early growth stage enlargement is exhibited in the hind limb bones as well.

Certain size-independent or proportionately distinct features appear in the skeleton of MCZ 8791 and some in OMNH 50268 but not in the skeletons of adult specimens of this taxon. Given these features appear only in the immature specimens of this taxon, they are considered ontogenetically informative. A morphological pattern of features appears in the skeleton of *B*. *feinbergorum* AMNH FR: 30556 that is similar to a pattern of morphological features that have been shown to be informative within the ontogeny of *D*. *antirrhopus*. From the similarity between these features in both *B*. *feinbergorum* and *D*. *antirrhopus* it can be determined that this conservative ontogenetic pattern is shared by at least two members of Dromaeosauridae.

As exemplified by MCZ 8791, there were no osteological obstacles or live weight limitations to flight capability within this early growth stage of *D*. *antirrhopus*. When compared to adult forelimb bones, the length of the juvenile manual II-2 phalanx is the longest proportionately exaggerated bone in the available forelimb bones of MCZ 8791. The MCZ 8791 manual II-2 phalanx ratios and dimensions indicate an especially long manual region of the forelimb. This would have enhanced the potential for juvenile dromaeosaurid flight.

## Supporting Information

S1 Character List List of 62 characters identified in MCZ 8791 from Turner et al. (2007) Appendix 2 Character List [5]. (DOCX)
