# Supplementary material for: Morphological Variations within the Ontogeny of Deinonychus antirrhopus (Theropoda, Dromaeosauridae)
Source: PLoS One. 2015 Apr 15;10(4):e0121476. doi: 10.1371/journal.pone.0121476 (PMC4398413; doi:10.1371/journal.pone.0121476)
Supplement: S1 Character List — (DOCX) [file pone.0121476.s001.docx]

S1_Character List

62 identified characters in MCZ 8791. Characters cited from: Turner et al. (2007) 182 BULLETIN AMERICAN MUSEUM OF NATURAL HISTORY NO. 371 APPENDIX 3 DATA MATRIX

For more complete character information go to the NEXUS file of the complete dataset which is available on Morphobank (O’Leary and Kaufman, 2007; http://www.

morphobank.org or <http://morphobank.org/permalink/?660>).

Character numbers refer to Turner et al. (2007) numbering

Characters pertaining to MCZ 8791

**MCZ 8791 coded character state in bold.**

Character 27: Pronounced, round accessory antorbital fenestra

0: absent

**1: present, fenestra occupies less than half of the depressed area between the anterior margins of the antorbital fossa and antorbital fenestra**

2: present, fenestra large and takes up most of the space between the anterior margins of the antorbital fenestra and fossa.

Character 28: Accessory antorbital fossa

0: situated at rostral border of antorbital fossa

**1: situated posterior to rostral border of fossa**

Character 29: Tertiary antorbital fenestra (fenestra promaxillaris)

0: absent

**1: present**

Character 77: Articular

0: without elongate, slender medial, posteromedial, or mediodorsal process from retroarticular process

**1: with process**

Character 78: Retroarticular process

**0: short, stout**

1: elongate and slender

Character 82: Maxilla

**0: toothed**

1: edentulous

Character 83: Maxillary and dentary teeth

0: serrated

**1: some without serrations anteriorly (except at base in S. mongoliensis)**

2: all without serrations

Character 84: Dentary and maxillary teeth

**0: large**

1: small (25–30 in dentary)

Character 86: Serration denticles

0: large

**1: small**

Character 87: Serrations

**0: simple, denticles convex**

1: distal and often mesial edges of teeth with large, hooked denticles that point toward the tip of the crown

Character 88: Teeth

0: constricted between root and crown

**1: root and crown confluent**

Character 100: Cervical centra

**0: with one pair of pneumatic openings**

1: with two pairs of pneumatic openings

Character 101: Cervical and anterior trunk vertebrae

**0: amphiplatyan**

1: opisthocoelous

2: at least partially heterocoelous

Character 103: Parapophyses of posterior trunk vertebrae

0: flush with neural arch

**1: distinctly projected on pedicels**

Character 106: Cervical vertebrae pneumaticity

0: absent

**1: present**

Character 115: Free caudal vertebrae

**0: with distinct transition point, from shorter centra with long transverse processes proximally to longer centra with small or no transverse processes distally**

1: vertebrae homogeneous in shape, without transition point

Character 117: Anterior caudal centra

0: tall, oval in cross section

**1: with boxlike centra in caudals I–V**

2: anterior caudal centra laterally compressed with ventral keel

Character 118: Neural spines of caudal vertebrae

**0: simple, undivided**

1: separated into anterior and posterior alae throughout much of caudal sequence

Character 119: Neural spines on distal caudals

0: form a low ridge

**1: spine absent**

2: midline sulcus in center of neural arch

Character 134: Posterolateral surface of coracoid ventral to glenoid fossa

0: unexpanded

**1: posterolateral edge of coracoid expanded to form triangular subglenoid fossa bounded laterally by enlarged coracoid tuber**

Character 135: Scapula and coracoid

**0: separate**

1: fused into scapulacoracoid

Character 136: Coracoid in lateral view

0: subcircular, with shallow ventral blade

**1: subquadrangular with extensive ventral blade**

2: shallow ventral blade with elongate posteroventral process

3: height more than twice width—coracoid strutlike

Character 137: Scapula and coracoid

0: form a continuous arc in posterior and anterior views

**1: coracoid inflected medially, scapulocoracoid L shaped in lateral view**

Character 138: Glenoid fossa faces

0: posteriorly or posterolaterally

**1: laterally**

Character 143: Distal articular surface of ulna (dorsal condyle and dorsal trochlea in birds)

**0: flat**

1: convex, semilunate surface.

Character 144: Proximal surface of ulna

0: a single continuous articular facet

**1: divided into two distinct fossae (one convex, the other concave) separated by a median ridge**

Character 192: Medial surface of proximal end of fibula

**0: concave along long axis**

1: flat

Character 193: Deep oval fossa on medial surface of fibula near proximal end

**0: absent**

1: present

Character 195: Tibia, cnemial crest(s)

**0: lateral crest only**

1: lateral and anterior crests developed.

Character 204: Ungual and penultimate phalanx of pedal digit II

0: similar to those of III

**1: penultimate phalanx highly modified for extreme hyperextension, ungual more strongly curved and significantly larger than that of digit III**

Character 214: Radius and ulna

**0: well separated**

1: with distinct adherence or syndesmosis distally

Character 221: Posterior edge of coracoid

**0: not or only shallowly indented below glenoid**

1: deeply notched just ventral to glenoid, glenoid lip everted

Character 222: Retroarticular process

**0: points caudally**

1: curves gently dorsocaudally

Character 228: Flexor heel on phalanx II-2

0: small and asymmetrically developed only on medial side of vertical ridge subdividing proximal articulation

**1: heel long and lobate, with extension of midline ridge extending onto its dorsal surface**

Character 237: Dorsal displacement of accessory (maxillary) fenestra

0: absent

**1: present**

Character 239: Accessory antorbital (maxillary) fenestra recessed within a shallow, caudally or caudodorsally open fossa, which is itself located within the maxillary antorbital fossa

0: absent

**1: present**

Character 247: Anterior and posterior denticles of teeth

0: not significantly different in size

**1: anterior denticles, when present, significantly smaller than posterior denticles**

Character 248: Maxillary teeth

0: almost perpendicular to jaw margin

**1: inclined strongly posteroventrally**

Character 302: Articular pneumaticity

0: absent

**1: present**

Character 315: Thoracic vertebrae

**0: at least part of series with subround, central articular surfaces (e.g., amphicoelous/opisthocoelous) that lack the dorsoventral compression seen in heterocoelous vertebrae**

1: series completely heterocoelous

Character 316: Thoracic vertebrae, parapophyses

**0: rostral to transverse processes**

1: directly ventral to transverse processes (close to midpoint of vertebrae)

Character 317: Thoracic vertebrae, centra, length, and midpoint width

**0: approximately equal in length and midpoint width**

1: length markedly greater than midpoint width

Character 318: Thoracic vertebrae, lateral surfaces of centra

**0: flat to slightly depressed**

1: deep, emarginated fossae

2: central ovoid foramina

Character 339: Scapula and coracoid articulation

0: pit-shaped scapular cotyla developed on the coracoid, and coracoidal tubercle developed on thescapula (‘‘ball and socket’’ articulation)

1: scapular articular surface of coracoid convex

**2: flat**

Character 340: Coracoid, procoracoid process

**0: absent**

1: present

Character 341: Coracoid, lateral margin

**0: straight to slightly concave**

1: convex

Character 342: Coracoid, dorsal surface

(= posterior surface of basal maniraptoran theropods)

**0: strongly concave**

1: flat to convex

Character 343: Coracoid, pneumatized

**0: absent**

1: present

Character 345: Coracoid, lateral process

**0: absent**

1: present

Character 346: Coracoid, ventral surface,

lateral intermuscular line or ridge

**0: absent**

1: present

Character 347: Coracoid, glenoid facet

**0: dorsal to, or at approximately same level as, acrocoracoid process/‘‘biceps tubercle’’**

1: ventral to acrocoracoid process

Character 349: Coracoid, n. supracoracoideus passes through coracoid

**0: present**

1: absent

Character 381: Ulna, cotylae

**0: dorsoventrally adjacent**

1: widely separated by a deep groove

Character 382: Ulna, dorsal cotyla convex

0: absent

**1: present**

Character 386: Radius, ventroposterior surface

**0: smooth**

1: with muscle impression along most of surface

2: deep longitudinal groove

Character 401: Manual digit II, phalanx 2, internal index process on posterodistal edge

**0: absent**

1: present

Character 416: Femur, ectocondylar tubercle and lateral condyle

**0: separated by deep notch**

1: form single trochlear surface

Character 417: Femur, posterior projection of the lateral border of the distal end,

continuous with lateral condyle

**0: absent**

1: present

Character 426: Tibia, distal-most mediolateral width

**0: wider than midpoint of shaft, giving distal profile a weakly developed triangular form**

1: approximately equal to shaft width, no distal expansion of whole shaft, although condyles may be variably splayed mediolaterally

Character 443: Radius width

**0: roughly half or greater than width of ulna**

1: less than half width of ulna

Character 458: Ulna, size of proximal cotylae

0: unequal, lateral (dorsal in birds) smaller

**1: equal**
